# Evidence of the active participation of women in the intergroup conflict based on the use of aggression and cooperation

**DOI:** 10.1038/s41598-023-45012-7

**Published:** 2023-10-18

**Authors:** José Antonio Muñoz-Reyes, Daniel Torrico-Bazoberry, Pablo Polo, Oriana Figueroa, Eugenio Guzmán-Lavín, Gabriela Fajardo, Nohelia Valenzuela, Montserrat Belinchón, Carlos Rodríguez-Sickert, Miguel Pita

**Affiliations:** 1 Laboratorio de Comportamiento Animal y Humano, Centro de Investigación en Complejidad Social, Facultad de Gobierno, Universidad del Desarrollo, Santiago, Chile; 2 Facultad de Administración y Economía, Universidad de Santiago, Santiago, Chile; 3 Departamento de Biología, Universidad Autónoma de Madrid, Madrid, Spain

**Keywords:** Psychology, Human behaviour

## Abstract

Intergroup conflict has been a persistent aspect of human societies since the emergence of our species. Various researchers have proposed that competition between groups has acted as a key selective force throughout human evolutionary history. Such intergroup competition for limited resources exacerbated the expression of intergroup aggression and intragroup cooperation. Furthermore, it would have a sexual dimorphism, with men demonstrating increased sensitivity to conflict threats—in order to maximize reproductive opportunities—, while women generally reject from active engagement in intergroup conflict. In the present study, we conducted behavioral experiments under controlled laboratory conditions to measure cooperation and aggression from using virtual games, specifically the Public Good Games and the Point Subtraction Aggression Paradigm, in a sample of 541 participants. We created control and experimental intergroup competition scenarios, where aggression and cooperation were necessary to increase monetary rewards. Our results shows that men modulate aggression and cooperation in the presence of intergroup conflict. In addition, our data also reveals that women cooperate more than men and display heightened levels of cooperation and aggression when confronted with intergroup conflict. These findings prompt a reevaluation of current functional theoretical models concerning the role of women in intergroup conflict and suggest that the dynamics of human aggression and cooperation may be more nuanced than previously believed.

## Introduction

Aggressive conflict between groups of human beings has been ubiquitous in societies dating back to the dawn of our species^1^. Numerous authors have proposed that between-group competition has been one of the main selective forces driven human evolution, even surpassing defense against other predatory species^2–5^. In this sense, the monopolization of physical resources and access to reproductive partners were the main axes on which the motivation to compete between groups was built, despite the devastating effects of being defeated^5^. In fact, from different fields as evolutionary psychology and primatology, it has been suggested that the exacerbated cooperative behavior observed in the human species would be, in large part, the product of a long history of competition between rival groups^4,6,7^. In this regard, functional models have been focused on the central role of men in the intergroup conflict^7^, which has been supported in several studies (e.g.^8–10^). However, the role of women in the intergroup conflict has been poorly studied.

Functional perspectives to understand sex differences in intergroup conflict are constructed from Trivers' theory of parental investment within the framework of sexual selection^11^, which highlights the asymmetry between male and female in parental investment and reproductive success. Accordingly, males have a greater variance in their reproductive success since they depend on optimizing access to females due their lower mandatory minimal parental investment. In contrast, females experience less variability in reproductive success, as they are more dependent on their mate’s quality, whether they are good providers or bearers of good genes due their higher mandatory minimal parental investment. Consequently, males generally engage in more intensive intrasexual competition to secure access to reproductive partners, while females would be more selective, and engage in less intense intrasexual competition^12^. This has generated many of the physical and behavioral differences that we currently see between both sexes in humans^3^. For example, in upper-body strength men are generally 90% stronger than women^13–15^, which is one of the most significant factors driving the differential ability to inflict costs to a rival^16^. According to behavior, differences are present in the tendency to use direct aggression and, especially in physical aggression (e.g.,^17,18^) in men. In this context, men would be more inclined to compete, form coalitions, and engage in violent conflict with other groups. This tendency is driven by their heightened emphasis on reproductive success and the need to secure mates (see the Male Warrior Hypothesis in^7,8^). However, evidence from other research domains contradicts Trivers’ assumption that females act more selectively and passively^19–21^.

Women have been usually located within the intergroup conflict playing a passive role where they are considered the primary motivating resource for competition between men^8^. However, the little experimental evidence about women in intergroup conflict shows a similar behavioral pattern that of men in rejecting individuals from other groups (i.e., estimated from prejudiced behavior towards rivals^22^). This evidence suggests a more active role within the intergroup conflict for women, which has been interpreted as a defensive strategy to avoid infanticide and sexual coercion^8^, although it is obvious that both sexes could be benefited from the monopolization of resources. In this sense, men’s participation in intergroup conflicts such as wars is predominant^23^, however, studies have shown that women, although in a lesser extent than men, tend to be part in conflicts. For instance, in the United States, the number of female war veterans has increased to 1.7 million in 2006, according to^24^. This trend was already evident during the Gulf War (1991–1992), when women constituted 11% of the allied active-duty personnel, and approximately 4% of those killed in combat were female^25^. These statistics, in addition to archaeological evidence (e.g.,^26,27^), suggest that women participation in intergroup conflicts is a real phenomenon, but that has been dismissed historically^28^. Accordingly, considering the available evidence, it is reasonable to propose that women's motives for actively participating in intergroup conflicts could be oriented to obtain limited resources. In this sense, food acquisition is fundamental for female reproductive success^11,29^. In fact, primatological studies show that intergroup competition for resources plays a critical role in shaping female gregariousness^30^. As a result, women could be motivated to participate in intergroup conflict—in addition to avoid infanticide and sexual coercion—, to access more or better resources which would increase their fitness.

There is a lack of studies that, under controlled conditions estimates the role of women in the intergroup conflict. In this sense, there are overlooked two sex-dependent factors that could be relevant to understanding it: the differences in aggressive mechanisms employed by men and women, and the tendency to cooperate based on group composition. Accordingly, existing research on intergroup conflict^31,32^ has predominantly focused on direct physical aggression, which is a form of aggression more commonly utilized by men^17^. These studies have demonstrated that men exacerbate aggression when this is directed to outgroup members (e.g.,^9^). However, this emphasis on direct physical aggression may have inadvertently limited our understanding of the diverse ways women contribute to and participate in intergroup conflicts, for example, giving support to their more physical stronger partners. In this sense, studies investigating ingroup cooperation often not consider the influence of group sex composition (^10,33^; for an exception, see^7^). Newly these studies have demonstrated a highly sensitivity of men to intergroup conflict from the increase of ingroup cooperation (e.g.,^7,9^, etc.). These approach falls short in revealing the behavioral architecture of the group outside of typical male behavior. For more than 30 years, both social psychology (e.g.,^34,35^) and evolutionary psychology have shown sex differences in the use of aggressive mechanisms^17^. According to evolutionary psychology, sex differences in aggression are the product of selective pressures that have changed the psychobiological underpinnings of behavior. As a result, rather than engaging in direct physical aggression, women often choose to use intermediaries to cause harm to a third party^18,36,37^. This tendency can be attributed to the heightened vulnerability of the female body to the detrimental effects of physical aggression on reproductive capacity^36,38^. Concerning cooperation there is evidence indicating that men cooperate more with individuals of the same sex^39,40^ even with those of lower status (e.g.,^41^), whereas women exhibit higher rates of cooperation in mixed-sex interactions^42^. This pattern indicates that women are skilled at forming tactical partnerships with men. Consequently, the position of women within the intergroup conflict must be delineated considering the typical aggressive mechanisms of each sex and the capacity of women to deploy robust mechanisms of cooperation with men, as stated above. Thus, addressing these two aspects may shed light on the active and offensive roles women can play in intergroup conflict, aspects that have been largely overlooked to date.

In the present study, we seek to replicate previous results centered in the modulation of cooperation and aggression under intergroup conflict in men (theorized in the Male Warrior Hypothesis by^7^). But, more interestingly, we want to understand women's role in intergroup conflict from the use of sex specific mechanisms of aggression and cooperation. We expect that as occurs with men, women will increase intragroup cooperation and intergroup aggression in presence of intergroup conflict, probably because women have motivation to use intergroup conflict as a scenario to gaining access to limited resources. We have assessed with an experimental design the ingroup cooperation through public good games and intergroup aggression from the use of a modified version of the point subtraction aggression paradigm (PSAP), which assessed direct and indirect aggression, taking into account group composition and under two experimental conditions, an intergroup conflict scenario and a control condition.

Concerning aggression, we have two set of predictions: First, we predict sex-based differences in the type of aggression displayed. Specifically, men will exhibit more direct aggression than women, whereas women will display more indirect aggression compared to men. Second, we predict an increase in aggression during intergroup conflict, driven by a heightened level of the more common type of aggression in each sex—direct aggression in men and indirect aggression in women.

In terms of cooperation, our expectations are also twofold: First, we predict sex-based differences in cooperation depending on group composition. That is, men will cooperate more in unisexual groups compared to women, whereas women will cooperate more than men in mixed-sex groups. Second, we predict that in the context of intergroup conflict, men will increase cooperation in unisexual groups, while women will enhance cooperation in mixed-sex groups, as compared to a control scenario (see Behavioral Measurements).

## Methods

### Participants

Over a three-year period (2020–2022) we recruited a total of 541 participants aged between 18–45 years old, including 235 men and 306 women (mean ± SD: 25.13 ± 5.70 and 26.67 ± 6.49 years, respectively). Participants were recruited from online advertisements on the laboratory webpage and social media platforms (Instagram and Facebook). Each participant received $15.000 Chilean pesos (around $19 USD) for attending the experimental session. Additionally, they could receive an extra payment of up to another $15.000 Chilean pesos based on their performance in the games. Thus, participants could receive a maximum of $30.000 Chilean pesos. We chose to offer a substantial amount of money ($30.000 pesos represent 7.3% of the minimum monthly wage in Chile) to ensure participants interest and involvement in the experiments.

### Ethics committee authorization and ensuring anonymity

The Universidad del Desarrollo’s Ethics Committee approved the study’s protocols and data handling procedures. All processes were performed in conformity with the applicable guidelines and regulations. An informed consent form that explained the protocol and the confidentiality was given to each participant to read and sign. However, before participants signed it, at the beginning of the experimental session, one of the researchers read the consent aloud for them. Prior to taking part in the trial, each participant signed an informed consent form. To protect participant’s identities, we followed a conventional coding procedure.

### Group formation and the data collection procedure

**Figure 1:**
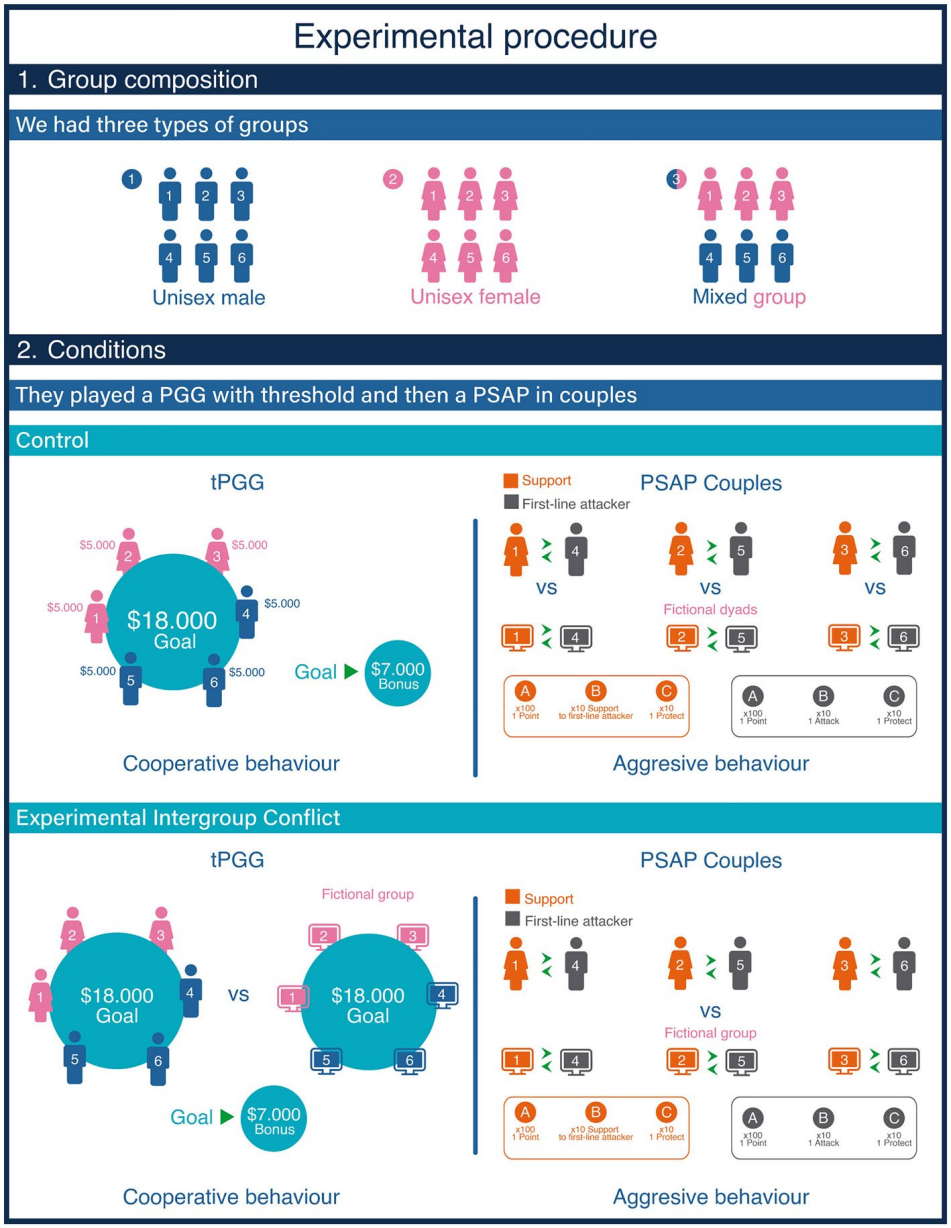
Group composition design.

The experiments took place at the “Laboratorio de Comportamiento Animal y Humano” of the Universidad del Desarrollo, Santiago de Chile. The laboratory has six experimental cabins with computers connected to a local network. These cabins were specifically designed to be isolated from visual and audio stimuli, ensuring that participants could not communicate with one another and could focus on the task at hand. In each experimental session, a group of six participants were assigned to either the intergroup competition scenario or the control scenario. Moreover, participants were also allocated to one of three group compositions: (a) all-male groups (or unisexual), (b) all-female groups, or (c) mixed-sex groups comprising three men and three women (Fig. 1). We privileged the formation of group of women as they were the main focus of this study.

After signing the informed consent, participants were randomly placed in individual cabins. In mixed-sex groups, men were assigned to one of the first three cabins, while women to the remaining cabins, in order to accommodate their distinct roles in the PSAP. At the beginning of each session, participants completed a sociodemographic questionnaire (i.e., sexual orientation, age, relationship status, place of origin and socioeconomic status). Subsequently, participants engaged in two economic games: (1) a threshold Public Goods Game (tPGG) and (2) a modified version of the PSAP, played in pairs. The tPGG was designed to elicit cooperative predispositions in the context of a larger group social dilemma, while the PSAP aimed to elicit aggressive inclinations at the individual level in the context of couple against couple interaction. The control scenario gauged both cooperative and aggressive dispositions, whereas the experimental scenario assessed how intergroup conflict modulates intercouple competition and intra-group cooperation respectively (Muñoz-Reyes et al. 2020). In the intergroup scenario, participants were informed that they were playing with another group with the same characteristics in terms of group composition, though in reality, they competed against a fictitious opponent (i.e., the game software). The same occurs for the control condition of the PSAP where participants must compete against fictitious couples located in another university.

### Behavioral measurements (see Fig. 1)

*Threshold public good game (tPPG)*: We applied the protocol used by Muñoz-Reyes et al.^9^, to measure individual cooperation within a group context. Participants played the tPGG on computers using the z-Tree software^43^. At the beginning of the game, each participant received $5,000 Chilean pesos and had the option to decide how much to contribute for the benefit of the group. They were informed that a bonus of $7,000 Chilean pesos would be awarded if the total group contribution surpassed $18,000 Chilean pesos, regardless of their individual contributions. However, if the group did not meet the threshold amount, no bonuses would be awarded, and participants would only retain the money they chose not to contribute.

In the control scenario, participants received no additional instructions beyond these basic rules. However, in the experimental scenario, they were informed that were playing with another group simultaneously and that the bonus would be given to the group that exceeded $18,000 Chilean pesos. Participants also were informed that in the event that both groups exceeded the threshold only the first group to make the decision would be granted the bonus. Since the competing group was fictitious the participants were rewarded with the bonus if they surpassed the threshold. This setup created a context of competition for a monopolizable resource. To ensure participants fully understood the game and scenario, an animated video was shown prior to the start of the game. Consistent with previous research^7,9^ participants played a practice round before the actual game commenced. The outcomes of the games were communicated at the end of all the experimental procedures to avoid any potential impact on subsequent games. Cooperation levels were measured based on each participant’s individual contribution to the group.

*The Point subtraction aggression paradigm (PSAP):* This is a widely recognized and reliable tool for measuring aggression, particularly in men and was first used by Cherek in the 1980s^9,44^. It is a computer game where participants play/compete against a fictitious opponent (but they did not know this information). The main objective is to get as many points as possible, which then are converted into real money at the end of the game. The participant’s score is displayed on the computer screen, and they are given three behavioral options that cannot be executed simultaneously. For our study, we employed a modified version of the game, in which individuals played in pairs against other fictional pairs (but did not handle this information). Each pair was composed of a “ front-line attacker” and a “supporter”. Only in mixed-sex groups, women were always assigned the supporter role (Fig. 1). As in the original game, individuals had three behavioral options that cannot be taken simultaneously, but one of the options (button B) differed for front-line attacker and supporters. Table 1 shows the main differences between the original version of the PSAP and the modified version that we used in this study.

**Table 1:** Shows the differences between the traditional PSAP and the modified PSAP.

Traditional PSAP	PSAP couples
Gaining points (button A): To obtain 1 gain point you must press the letter A 100 times 1 profit point equals 1000 Chilean pesos	Gaining points (button A): To obtain 1 gain point you must press the letter A 100 times 1 profit point equals 1000 Chilean pesos
Aggression (B button): To attack the opponent (fictitious) you must press the B key 10 times, which destroys one point of the opponent but does not accumulate them for your gain	Aggression (B button): Role 1: Front line attacker: this player can destroy points of the rival pair by pressing the letter B 10 times Role 2: Support: this player can boost the attack towards the rivals by pressing the letter B 10 times but as support for the attack role and not autonomously, that is, they were informed that they could not destroy rival points individually but only in pairs
Protection (C button): Individuals can protect themselves from the attack of rivals for a fixed period	Protection (C button): Individuals can protect themselves from the attack of rivals for a fixed period

*Gaining points (button A)*: Participants gain 1 point by pressing the “A” button 100 times. One point was equal to $1,000 Chilean pesos.

*Aggression (button B)*: In this option, “ front-line attacker” were informed that they could “destroy” points from the rival pair, but without gaining those points to their own score (i.e., destroying decreases the other player’s score without increasing one’s own). Participants were told that, by pressing the B button 10 times, they would destroy 1 point from the opponent pair's score. Additionally, they were informed that their rivals would receive the points that were taken from them, when the "front-line attackers" pressed the B button to destroy their points. Therefore, participants were in an asymmetrical condition with their rivals, what represents a constituent element of the game. The only effect of destroying points was to harm the opponent pair, without benefiting the player who pressed the button^9^. On the other hand, “supporters” were informed that they would not have the ability to directly destroy points from the rival pair. Instead, their role was to support potential attacks from the “ front-line attacker. More specifically, they were told that if they pressed the B key 10 times the next time their partner attacked (if it attacked at all) they would destroy the opponent's two points instead of one.

*Protection (button C)*: Particints were informed that their rivals could steal their points, so they were provided with an option to protect themselves by pressing the “C” button 10 times—which would protect them from possible attacks that could lead to the subtraction of their points during a fixed period.

A single 10-min round was conducted in both the control and experimental conditions. During these 10 min, the individuals could choose any of the three behaviors sequentially but not simultaneously. In other words, once a button was pressed, they needed to finish the sequence (i.e., the number of times that the selected button must be pressed to activate the different options of the PSAP) after choosing the same or other option (i.e., protection, aggression, gaining points). In the control condition, participants were informed that the points obtained by the pair would be added up and divided equally at the end of the game. In contrast, participants in the experimental scenario were told that they were part of a group competing against another group in a laboratory at another university (but it was a fictional couple). They were informed that each couple was going to be paired with another couple from the competing group (fictional group), and the winner would be the group that gained more points. The winning group would receive a bonus, equal to the points obtained by the losing group, which would be split evenly between the members of the winning group. The losing group would only receive the points obtained by each pair. As the competing group was fictitious, participants were always informed that they had won the match and were given a bonus equal to 50% of the points they obtained^9^(Muñoz-Reyes et al., 2020). To achieve greater ecological validity and consider the relevance of aggression in intergroup competition and intragroup status, we followed a strategy used by Geniole et al. (2017), in which participants are intensely provoked, that is, the (fictitious) rival attacked them very intensely, taking away points. Aggression was estimated by calculating the percentage of times the “B” button was pressed in relation to the total number of times all the buttons were pressed (see^9,44^). The aggression measured from first liners was interpreted as direct aggression whereas aggression measured from the supporters was interpreted as indirect aggression. The experimental scenario involves a conflict with an outgroup threat, with real potential consequences in terms of monetary payoffs, which the members of the group can collect by outcompeting the fictitious outgroup.

### Data analysis

To test our predictions regarding aggressive behavior, a full factorial general linear model was used with three factors: condition (intergroup conflict *vs*. control), sex (man *vs*. woman), and type of aggression (direct *vs*. indirect). Age was included as a control covariate, and the dependent variable was the aggression displayed by each participant in the PSAP. The two-way interaction between sex and type of aggression was used to test the first prediction, while the three-way interaction between sex, type of aggression, and condition, was used to test the second prediction. To avoid the possible confounding effect of mixed-sex group (in this configuration, we only have men performing direct aggression and women performing indirect aggression), we have repeated this analysis using only unisexual groups, (i.e., men and women performing direct and indirect aggression in same-sex groups). To test the two predictions concerning cooperative behavior, we fitted a full factorial general lineal model with three factors: condition (intergroup conflict *vs.* control), sex (man *vs*. woman), and group composition (unisexual vs. mixed). Age was also included as a control covariate, and the dependent variable was the contribution made by each participant in the public good game. The two-way interaction between sex and group composition was used to test the first prediction, while the three-way interaction between sex, group composition, and condition was used to test the second prediction.

For both models, we conducted pairwise comparisons with Bonferroni corrections when significant two-way or three-way interactions were present. We performed a sensitivity analysis that indicated we had 80% power to detect an effect size of η^2^ = 0.014, which is considered small. All analyses were carried out using IBM SPSS Statistics v25 software, and sensitivity analysis was conducted with G*Power 3.1.9.7. All analysis were two-tailed and our level of significance was set at α = 0.05.

## Results

Table 2 shows the mean, standard deviation, and sample size for cooperation in the tPGG, direct aggression and indirect aggression according to sex, condition, and group composition, the last only for cooperation measures.

**Table 2:** Mean, standard deviation (in parenthesis) and sample size for cooperation, direct aggression and indirect aggression according to sex, condition and group composition.

	Control				Intergroup competition			
	Men		Women		Men		Women	
	Unisexual	Mixed sex	Unisexual	Mixed sex	Unisexual	Mixed sex	Unisexual	Mixed sex
Cooperation	3027.04 (1195.02) N = 52	3492.33 (783.87) N = 58	3474.56 (803.06) N = 80	3534.81 (816.99) N = 59	3585.44 (1088.36) N = 63	3452.42 (926.94) N = 62	3631.22 (852.19) N = 109	3608.62 (717.34) N = 58

	Men		Women		Men		Women	
Direct aggression	0.0381 (0.0503) N = 84		0.0247 (0.0359) N = 43		0.0639 (0.0589) N = 95		0.0568 (0.0409) N = 58	
Indirect aggression	0.0621 (0.0468) N = 25		0.0623 (0.0645) N = 96		0.0718 (0.0597) N = 30		0.0747 (0.0661) N = 109	

Table 3 shows the model related to aggression. In relation to our first prediction, we did not find sex differences in the type of aggression employed since the two-way interaction between sex and type of aggression was not significant (F1,540 = 1.142, *p* = 0.286, η^2^ = 0.002). We found, instead, a main effect of the type of aggression (F1,540 = 15.152, *p* < 0.001, η^2^ = 0.028). Both men and women displayed more indirect aggression (M = 0.068, SE = 0.004) than direct aggression (M = 0.046, SE = 0.004). Regarding our second prediction, we did not find that men displayed more direct aggression and women more indirect aggression during intergroup conflict condition compared to control context since the three-way interaction was not significant (F1,540 = 0.028, *p* = 0.868, η^2^ < 0.001). However, we found a main effect of the condition (F1,540 = 12.667, *p* < 0.001, η^2^ = 0.023). Men and women display more aggression, both direct and indirect, during the intergroup conflict condition (M = 0.067, SE = 0.004) compared to the control condition (M = 0.047, SE = 0.004). When excluding mixed-sex groups, we obtained similar results. First, we did not find sex differences in the type of aggression employed since the two-way interaction between sex and type of aggression was not significant (F1,304 = 2.325, *p* = 0.128, η^2^ = 0.008). We found a main effect of the type of aggression (F1,304 = 9.461, *p* = 0.002, η^2^ = 0.031). Both men and women displayed more indirect aggression (M = 0.069, SE = 0.005) than direct aggression (M = 0.049, SE = 0.005) when playing with same-sex partners. Second, regarding our second prediction, we did not find that men displayed more direct aggression and women more indirect aggression during intergroup conflict condition compared to control context since the three-way interaction was not significant (F1,304 = 0.187, *p* = 0.666, η^2^ = 0.001). But similarly to the first analysis, we found a main effect of the condition (F1,304 = 12.514, *p* < 0.001, η^2^ = 0.041). Men and women display more aggression, both direct and indirect, during the intergroup conflict condition (M = 0.071, SE = 0.004) compared to the control condition (M = 0.047, SE = 0.005) when playing with same-sex partners.

**Table 3:** General lineal model of aggression in the PSAP according to sex, type of aggression and condition.

	F-value	*p* value	η^2^
Intercept	25.791	< 0.001	0.046
Sex	0.642	0.423	0.001
Type of aggression	15.152	< 0.001	0.028
Condition	12.667	< 0.001	0.023
Age	0.036	0.849	< 0.001
Sex * Type of aggression	1.142	0.286	0.002
Sex*Condition	0.151	0.698	< 0.001
Type of aggression * Condition	2.455	0.118	0.005
Sex * Type of aggression * Condition	0.028	0.868	< 0.001
Corrected model	4.758	< 0.001	0.067 (R^2^)/0.053 (R^2^_adj_)

**Figure 2:**
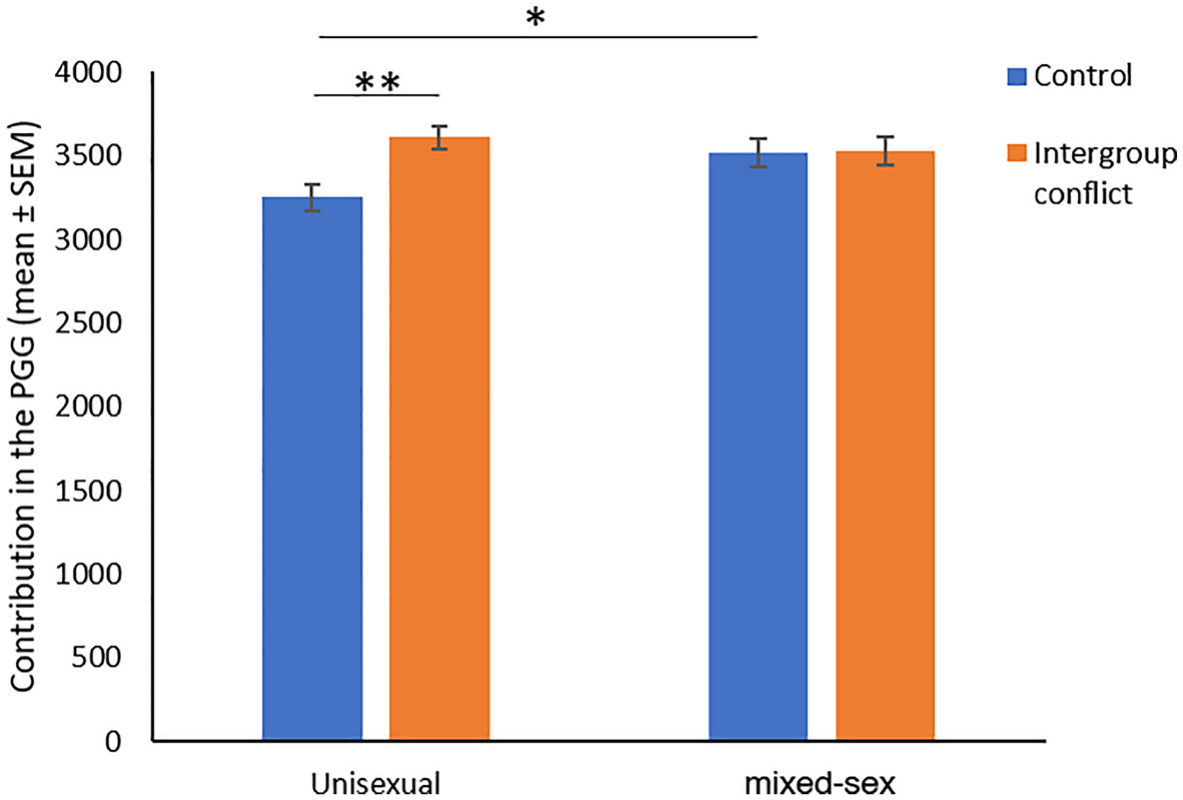
Contributions in the public good game according to group composition and treatment.

Table 4 shows the model related to cooperation. First, we did not find sex differences according group composition since the two-way interaction between sex and group composition was not significant (F1,541 = 0.870, *p* = 0.351, η^2^ = 0.002), but we found a sex differences as a main effect (F1,541 = 4.237, *p* = 0.040, η^2^ = 0.008). Women contributed more to the public good (M = 3558.492, SE = 53.30) than men did (M = 3394.21, SE = 59.06) regardless of group composition. Considering the presence of intergroup conflict, we only found a statistical trend in the three-way interaction between sex, group composition and context (F 1,541 = 2.724, *p* = 0.099, η^2^ = 0.005). Alternatively, if we focus in the two-ways interactions, we found a significant interaction between group composition and context (F1,541 = 4.674, *p* = 0.031, η^2^ = 0.009). Post-hoc analysis with Bonferroni correction (Fig. 2) showed that individuals contribute more in the intergroup conflict condition (M = 3609.21, SE = 71.18) compared to control condition (M = 3251.46, SE = 80.11) in the unisexual group composition (mean differences = 357.75, SE = 107.167, *p* = 0.001) but not in the mixed-sex group composition (mean differences = 14.86, SE = 116.91, *p* = 0.899). In addition, individuals contribute more in mixed-sex groups (M = 3514.94, SE = 83.17) compared to unisexual groups (M = 3251.46, SE = 80.11) but only in the control condition (mean differences = 263.47, SE = 115.47, *p* = 0.023) since in the intergroup conflict condition no differences were found (mean differences = − 79.41, SE = 108.71, *p* = 0.465).

**Table 4:** General lineal model of contributions in the public good game according to sex, group composition and condition.

	F-value	*p* value	η^2^
Intercept	391.664	< 0.001	0.424
Sex	4.237	0.040	0.008
Group composition	1.347	0.246	0.003
Condition	5.520	0.019	0.010
Age	0.920	0.338	0.002
Sex * Group composition	0.870	0.351	0.002
Sex*Condition	0.792	0.374	0.001
Group composition * Condition	4.674	0.031	0.009
Sex * Group composition * Condition	2.724	0.099	0.005
Corrected model	2.419	0.014	0.035 (R^2^)/0.021 (R^2^_adj_)

## Discussion

In the present study, we seek to understand how intergroup conflict modulates the expression of aggression and cooperation in both sexes. While we obtained partial support for our predictions, our results indicate that intergroup conflict exacerbates the expression of direct and indirect aggression in both sexes. Furthermore, women tend to be more cooperative than men, and cooperation was exacerbated in intergroup conflict but only in unisexual groups of men and women. Overall, our study contributes to a better understanding of the roles of men and women in intergroup conflict.

Our first set of prediction focused on aggression and was divided into two parts. In line with prior research (e.g.,^17,45^), we expected sex differences in the utilization of aggression. Observations in natural settings have revealed distinctions in type of aggression employed, with men consistently exhibiting a greater propensity for physical aggression^46^. This can be attributed to the higher costs associated with engaging in physical aggression for women^47^. However, contrary to our expectations, we did not observe any differences in the types of aggression exhibited by men and women, as there was no interaction between sex and aggression type. Given that sex differences in aggression have been consistently reported in previous research (e.g.,^18,48–50^), we can speculate that the expression of sexual dimorphism we anticipated need additional elements that were not incorporated into our experimental design that are present in natural contexts. For instance, our design did not allow participants to choose between first-liners (direct attack) and supporters roles; instead, they were forced to engage in a specific type of aggression due to their assigned role within the experiment. Moreover, in our experiment, men were only assigned to display indirect aggression in unisexual groups, while women assumed this role in both unisexual and mixed-sex groups. However, when we analyzed only unisexual groups, the pattern of results was maintained. Thus, it is possible that sex differences in the use of indirect aggression may only emerge if men reduce their employment specifically when interacting with women or if both sexes can choose between using direct or indirect aggression. Another explanation is based on the magnitude of the perceived threat and the inability to assess rivals in our experimental setup. Sex differences in aggression have been found to vary in magnitude depending on the level of threat^47^. However, in our study, participants could not observe their rivals, and the controlled laboratory environment lacked the contextual cues present in natural settings. This absence of contextual information could influence the types of aggression exhibited by both sexes under the experimental conditions. The relevance of this observation relies in the fact that specific signals, such as upper trunk strength or formidability, play a crucial role in determining whether (or not) to initiate direct aggressive interactions^51^. In our study, individuals were unable to assess their rivals in terms of physical strength, which could have influenced the expression of direct aggression for both sexes. Consequently, to further examine this intriguing finding, it is essential to employ new methodologies that can validate the absence of sexual dimorphism in aggression, at least in the context of virtual games conducted under controlled laboratory conditions.

In the second part of our prediction, we expected an increase in aggression in both sexes within the intergroup conflict scenario. Specifically, we expected more direct aggression in men and increased indirect aggression in women. As previously noted, we did not find sex differences in the expression of aggression. However, we did observe a context effect on aggression, i.e., both sexes exacerbate their direct and indirect aggressive strategies during intergroup conflict, illustrating that they are sensitive to intergroup conflict situations. Furthermore, this finding is consistent with previous evidence of women participating in conflicts (e.g.,^20,25,52^). In this regard, our results suggest that it is probable that both sexes would pursue the acquisition of resources, which is interesting because although the participation of women in conflicts such as war is lesser than men, our results indicate that when women have to confront to another group, they can assume an active role. In our game, women were not suffering the risk of infanticide or sexual coercion; the incentive consisted of a relevant monetary reward. We conclude that, as occurs with men, the possibility to obtain a valuable limited resource is an incentive for increasing aggression. These results contribute to reevaluating the roles and motivations underlying women’s expression of aggression in intergroup conflict situations.

Our set of predictions regarding cooperation, were also divided into two parts. In the first one, we expected sex differences in cooperation according to the group composition. In this sense, women were expected to be more cooperative than men in mixed-sex groups while men were expected to be more cooperative than women in unisexual groups. From a functional approach, it is expectable that men will be more prone to cooperate with same-sex group members since the conformation of coalitions of men has been proposed as a critical factor for the success of the group^5,7,8,53^. In contrast, previous evidence demonstrates that women cooperate more than men in mixed groups^42^, and actively compete to form bonds with higher-status men^54^. However, although there were theoretical reasons to expect this dimorphic pattern of behavior, our results indicate that group composition is irrelevant to explaining cooperation in both sexes, as women were consistently more cooperative than men, regardless of group composition. These results align with a recent meta-analysis that found no effect of sex composition on cooperation rates, but contrast with it since we found overall differences in cooperation between sexes^55^. Therefore, our results suggest that factors other than group composition likely explain the tendency to cooperate in men and women, with women being more cooperative overall.

In the second part of this prediction, we assessed cooperation considering sex differences but now in the presence of intergroup conflict. Unexpectedly, we found that, independently of sex, unisexual groups increased cooperation under intergroup conflict. In addition, mixed-sex groups were not sensible to intergroup conflict. Although there were no sex differences in this behavior, different functional explanations can explain it for each sex. For men, our results confirm the previous research of men in the functional approach, specifically under the theoretical framework of the male warrior hypothesis (see van^9,56^), i.e., men increase intragroup cooperation in the presence of intergroup conflict, to be compared with the control condition, probably because men are seeking to robustness alliances with other men in the presence of an external menace. In contrast, for women our explanation is quite different. In this sense, women are known to be more fearful than men of being socially excluded by other women^57,58^, this is a very effective and typical intrasexual competitive mechanism of this sex. As noted by^54^, in situations where women are surrounded by unrelated same-sex peers, as occurred in our experimental design, they may be more likely to form coalitions quickly to avoid the potential threat of social exclusion, which could be devastating in a context of intergroup conflict. Therefore, we propose they are sensible to intergroup conflict where cooperation is needed to construct internal alliances in the confrontation with the exogenous group.

Following with prediction two and regarding mixed-sex groups, as we have mentioned, no differences between control and intergroup scenario were found. However, in the control condition mixed-sex groups cooperate more than unisexual groups, and in the intergroup conflict condition there were no differences between unisexual and mixed-sex groups. This suggests that, irrespective of the scenario, mixed-sex groups seem to cooperate at a similar rate as unisexual groups during intergroup conflict situations. One plausible explanation for those relatively high levels of cooperation observed in the mixed-sex groups could be attributed to the presence of an intragroup mating scenario. It is known that the presence of women tends to enhance competitive altruism in men^59,60^. Despite the fact that contributions were anonymously, group performance was informed at the end of the experimental procedure, allowing participants to exchange details about their donations. Consequently, it is conceivable that mating motives for men may overshadow the effects of the intergroup competition context, resulting in high levels of cooperation in both control and intergroup conflict scenarios. The concept of competitive altruism among women has been discussed in the literature, but studies predominantly focus on men^59,61–63^ but this factor may contribute as well to the pattern found. However, further studies would be required to better explain and understand why groups composed by men and women seem not to be sensitive to the presence of an intergroup conflict scenario. Nevertheless, it is noteworthy that our study detected a strong sensitivity to intergroup conflict in same-sex groups for both men and women. Surprisingly, and contrary to our initial predictions, this was also observed in groups of women. Future studies are needed to reply to these findings.

Within the limitations of this study, we can mention that participants could not choose their role within the game, as they were assigned to one or another condition. In addition, the result obtained in our study i.e., a lack of sex differences in the use of aggressive behavior, indicate that it could be relevant for future studies to include mixed groups where women occupy the role of the front-line attack. We have not considered this condition in the current study since, as we stated before, the objective was to observe if women were sensitive to intergroup conflict in their aggressive behavioral mechanism, beyond the composition of the group (mixed or unisexual). Another elements for future studies could be to include psychological measures such as risk aversion or dark triad assessments. These additions could shed light on other aspects of the psychology of men and women in intergroup conflict situations. Additionally, it would be beneficial to allow participants the possibility of evaluating their rivals. While incorporating such a feature might be challenging in terms of experimental design, it could yield valuable insights into the strategies employed by individuals during intergroup conflicts. It is relevant to highlight a group of studies^64–66^ which proposed that ingroup love over outgroup menace is the real motivation that could explain sex differences in cooperation, with men preferring ingroup cooperation versus outgroup cooperation beyond of the presence of intergroup conflict. These studies applied a treatment to encourage group identity from the minimal group paradigm^66^. We propose that future studies integrate this methodological tool to discuss the possibility that our results could be explained by a weaker ingroup love in men’s participants due to the lack of this treatment. Ultimately, our findings emphasize the need to increase the complexity of experimental design to fully understand how intergroup conflict modulates human behavior.

Our study has uncovered new evidence suggesting that women exacerbate aggression in intergroup conflict scenarios. In the case of cooperation, we found it to be higher in women than men, with both sexes increasing cooperation in same-sex groups in intergroup conflict scenarios. Concerning aggression, both sexes increase direct and indirect aggression in the intergroup conflict scenario at least when interacting with same-sex partners. These results are interesting since they open the possibility that women will be sensible to conflict beyond the view of men being primarily interested and sensitive to participate in intergroup conflict actively. In the same sense, recent studies have proposed a change in the expected pattern of behavior for other resource acquisition behavior in traditional societies, such as hunting (e.g.,^67,68^). This study invites to enrich the current theoretical models by looking for new evidence about the roles of both sexes in intergroup conflict.

## Data Availability

Data for the study “Women in the intergroup conflict. Evidence of active participation from the use of aggression and cooperation” are available at Open Science Framework OSF: osf.io/u86zx.
